# Status epilepticus affects the gigantocellular network of the pontine reticular formation

**DOI:** 10.1186/1471-2202-10-133

**Published:** 2009-11-13

**Authors:** Péter Baracskay, Viola Kiglics, Katalin A Kékesi, Gábor Juhász, András Czurkó

**Affiliations:** 1 Laboratory of Proteomics, Institute of Biology, Eötvös Loránd University, H-1117 Budapest, Hungary; 2 Department of Physiology and Neurobiology, Eötvös Loránd University, H-1117 Budapest, Hungary; 3 Institute of Medical Chemistry, University of Szeged, H-6720 Szeged, Hungary

## Abstract

**Background:**

The impairment of the pontine reticular formation (PRF) has recently been revealed to be histopathologically connected with focal-cortical seizure induced generalized convulsive *status epilepticus*. To elucidate whether the impairment of the PRF is a general phenomenon during *status epilepticus*, the focal-cortical 4-aminopyridine (4-AP) application was compared with other epilepsy models. The presence of "dark" neurons in the PRF was investigated by the sensitive silver method of Gallyas in rats sacrificed at 3 h after focal 4-AP crystal or systemic 4-AP, pilocarpine, or kainic acid application. The behavioral signs of the developing epileptic seizures were scored in all rats. The EEG activity was recorded in eight rats.

**Results:**

Regardless of the initiating drug or method of administration, "dark" neurons were consistently found in the PRF of animals entered the later phases of *status epilepticus*. EEG recordings demonstrated the presence of slow oscillations (1.5-2.5 Hz) simultaneously with the appearance of giant "dark" neurons in the PRF.

**Conclusion:**

We argue that the observed slow oscillation corresponds to the late periodic epileptiform discharge phase of *status epilepticus*, and that the PRF may be involved in the progression of *status epilepticus*.

## Background

*Status epilepticus *is an emergency in clinical practice, but the detailed mechanism of its pathology is less well known. In fact, very few studies have examined the clinical consequences of *status epilepticus *as a single morbidity [1], although a progressive sequence of electroencephalographic (EEG) changes during generalized convulsive *status epilepticus *(GCSE) has been described [2]. GCSE typically begins as partial-onset *status epilepticus *and secondarily generalizes [3].

Recently, by combination of EEG monitoring and a sensitive silver method of Gallyas [4-7], we discovered two "dark" neuron populations that may be of crucial importance in generalization of epileptic seizures and the progression of *status epilepticus *[8]. Histopathologically, epileptic seizures can produce "dark" morphological change in neurons [9], and these affected neurons are selectively and "spectacularly" visualized by the Gallyas silver method [10,11]. In our study, epileptic seizures induced by focal-cortical application of K^+ ^channel blocker 4-aminopyridine (4-AP) developed into *status epilepticus *with a slow oscillation (~1.5 Hz) at 6 h, which was correlated with the appearance of both "dark" neurons in the pontine reticular formation (PRF) and "dark" interneurons in the hippocampus [8]. The temporal profile of neuronal injury in the hippocampus is well described [6] and it is known that spontaneous seizures preferentially injure interneurons in the hippocampus [4]. Therefore, in this study we focused on the PRF.

It is not known whether the giant neurons of the PRF are involved in the progression of *status epilepticus*, although electrophysiological studies have suggested that the PRF may participate in generation and maintenance of the epileptic state [12-15]. Nevertheless, experimental histopathology experiments have not investigated the PRF for the presence of "dark" neurons following pilocarpine- or kainic acid-induced *status epilepticus *[6].

In this study, we investigated the PRF for "dark" neurons 3 hours after the induction of epileptic seizures. Focal-cortical application of 4-AP was compared with systemic administration of 4-AP, pilocarpine or kainic acid. "Dark" neurons were found in the PRF in all prolonged *status epilepticus *cases. It is also known from previous studies that brief seizures can confer tolerance against prolonged seizures and neuronal damage [7,16-19] for example, prior administration of 4-AP protects against kainic acid-induced neuronal cell death [20]. To investigate this, a cumulative approach was invoked to bring about *status epilepticus*. When the first attempt with 4-AP or pilocarpine failed to induced *status epilepticus*, we used kainic acid on the following day. Despite possible tolerance against cell injury, "dark" neurons were still consistently found in the PRF.

## Results

### Focal-cortical 4-AP crystal application

**Figure 1:**
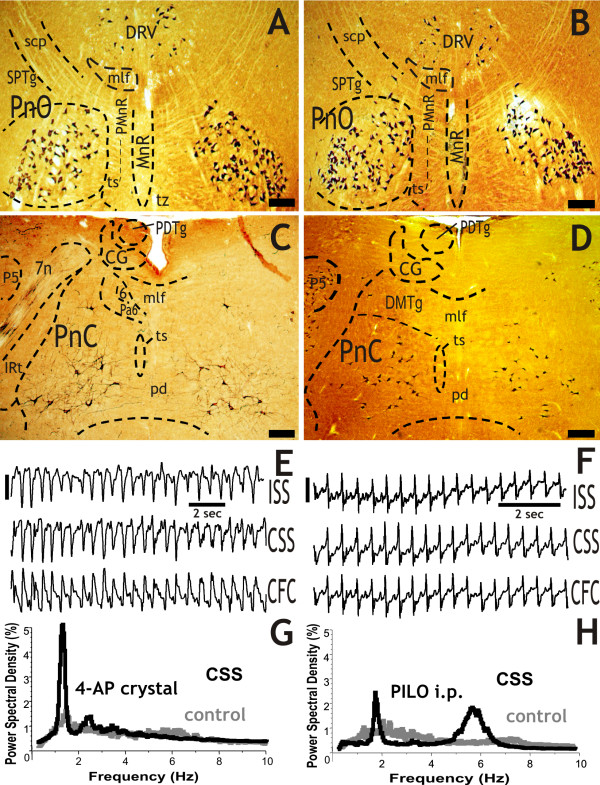
**Giant "dark" neurons (A, B, C and D) in the pontine reticular formation at 3 hours survival time after focal-cortical 4-AP crystal application (A and C) and systemic injection of pilocarpine (B and D)**. The PnO (A, B) and PnC (C, D) are symmetrically involved in both cases. The representative EEG periods demonstrate the late phase of the generalized convulsive *status epilepticus *(E and F). Three hours after the 4-AP application, the power spectral density (PSD) graph of the one-hour-long EEG recording shows a high peak at the slow frequencies (1-1.5 Hz; G). After the pilocarpine injection, the PSD analysis also shows a peak at the slow frequencies (1.5-2 Hz) and an additional increase in the power at around 6 Hz (H). The control PSDs (gray lines) were calculated from 30 min EEG recordings before the treatment of each rat. Abbreviations: ISS and CSS: ipsi- and contralateral somatosensory cortex, CFC: contralateral frontal cortex, 6: abducens nucleus, 7n: facial nerve or its root, CG: central grey, DRV: dorsal raphe nucleus, ventral part, IRt: intermediate reticular nucleus, mlf: medial longitudinal fasciculus, MnR: median raphe nucleus, P5: peritrigeminal zone, Pa6: paraabducens nucleus, pd: predorsal bundle, PDTg: posterodorsal tegmental nucleus, PMnR: paramedian raphe nucleus, PnC: pontine reticular nucleus, caudal part, PnO: pontine reticular nucleus, oral part, scp: sup cerebellar peduncule, SPTg: subpeduncular tegmental nucleus, ts: tectospinal tract, tz: trapezoid body, 4-AP: 4-aminopyridine, PILO: pilocarpine. Scale bars: A, B, C and D = 400 μm, E and F = 200 μV.

**Figure 2:**
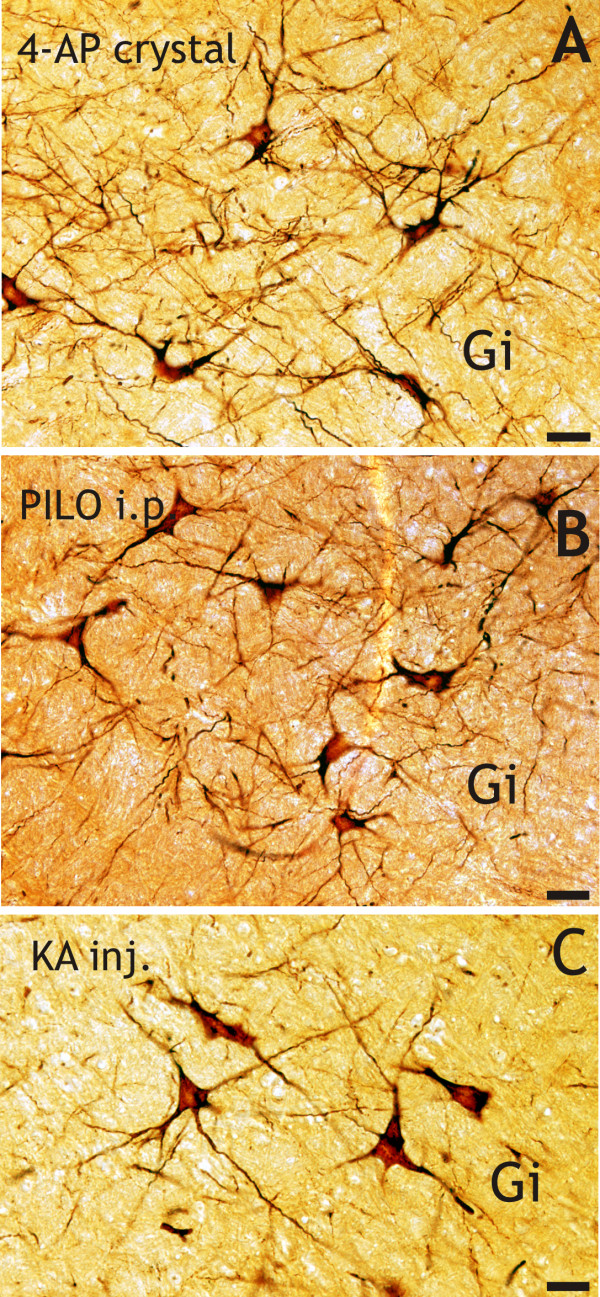
**The "dark" gigantocellular network in the ponto-medullar reticular formation at 3 hours survival time, after the administration of focal-cortical 4-AP crystal, pilocarpine or kainate**. The giant "dark" neurons are stained together with their long dendrites in the gigantocellular reticular field (Gi). Scale bars: A, B and C = 80 μm.

Out of the four rats examined histopathologically, three entered *status epilepticus *at 3 h. All three rats had giant "dark" neurons in their PRF (Table 1), both in the oral and caudal pontine reticular field (PnO, PnC; Figure 1A,C) and also in the medullary reticular field (Gi; Figure 2A). One animal did not enter *status epilepticus*, and no "dark" neurons were found in its PRF (Table 1).

**Table 1:** Comparison of focal-cortical and systemic models at 3 h

4-AP crystal (n = 4)					4-AP i.p. (n = 4)					Pilocarpine (n = 6)					Kainic Acid (n = 8)				
**i.d**.	**PRF** **DN**	**Bhv**. **scr**.	**SE**	**EEG**	**i.d**.	**PRF** **DN**	**Bhv**. **scr**.	**SE**	**EEG**	**i.d**.	**PRF** **DN**	**Bhv**. **scr**.	**SE**	**EEG**	**i.d**.	**PRF** **DN**	**Bhv**. **scr**.	**SE**	**EEG**

ac1	-	3	-		ai1	-	3	-		pi1	-	3	-		ka1_E_	+++	4	+	5
ac2	++	5	+		ai2	-	3	-		pi2	+	5	+		ka2_E_	+	3	-	3
ac3	+++	5	+		ai3	++	5	+		pi3	+++	5	+		ka3_E_	+++	4	+	5
ac4_E_	+++	5	+	5	ai4	+++	5	+		pi4_E_	+++	5	+	5	ka4_E_	-	3	-	2
										pi5	+++	5	+						
										pi6_E_	+++	5	+	5					
					
					ak1		2	-		pk1		2	-		ak1	++	5	+	
					ak2_E_		3	-	1	pk2		2	-		ak2_E_	+++	5	+	5
															pk1	+	4	-	
															pk2	++	5	+	

i.d.: animal i.d., PRF DN: number of „dark” neurons in the PRF, Bhv.scr.: behavioral scoring with Racine Scale; SE: if *Status Epilepticus *developed (+) or not (-), EEG: Treiman's EEG phases,

The relative numbers of "dark" neurons averaged from five 60-μm sections of the pontine and medullar reticular formation are expressed with a semi-quantitative grading scale as follows: +: 1-5 "dark" neurons; ++: 5-15 "dark" neurons, +++: 15-30 "dark" neurons.

### Systemic injections of 4-AP

Out of the four rats examined, two entered *status epilepticus *at 3 h, and they had giant "dark" neurons in their PRF (Table 1), in the PnO, PnC and in the mesencephalic and medullary reticular field (Gi). Two animals did not enter *status epilepticus*, and no "dark" neurons were found in their PRF (Table 1).

### Systemic injections of pilocarpine

Five of the six rats examined entered *status epilepticus *at 3 h, and all five had giant "dark" neurons in their PRF (Table 1), in the PnO, PnC (Figure 1B, D; and see Additional file 1B) and Gi (Figure 2B). One animal did not enter *status epilepticus*, and no "dark" neurons were found in its PRF (see Additional file 1A; Table 1).

### Systemic injections of kainic acid

Two out of the four rats examined entered *status epilepticus *at 3 h, and both had giant "dark" neurons in their PRF (Table 1). The two other animals did not enter *status epilepticus*, but they reached Stage-3 of the behavioral scoring: forelimb clonus and "wet dog shakes". In one of these animals 4-5 "dark" neurons were found in its PRF, the other had none (Table 1).

### Systemic injections of kainic acid as a second treatment

**Figure 3:**
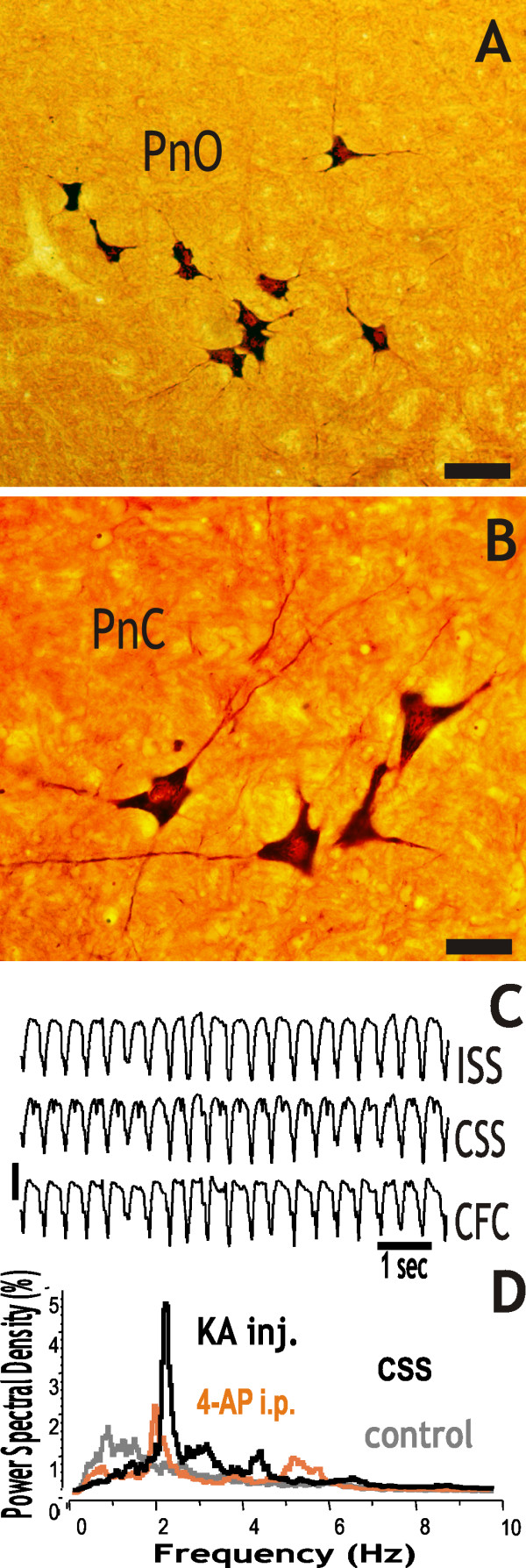
**Giant "dark" neurons in the pontine reticular formation (A and B) at 3 hours survival time after intramuscular injection of kainic acid (KA)**. The Golgi-like staining highlights the giant "dark" neurons of the oral pontine reticular field (PnO; A) and caudal pontine reticular field (PnC; B). The representative EEG period demonstrates the generalized convulsive status epilepticus (C). On the one-hour PSD graphs, there is a smaller peak at 2 Hz after the 4-AP injection a day before the kainic acid treatment (red line), while there is a prominent, high peak in the PSD at 2-2.5 Hz at 3 hours after the KA (D). The control PSD (gray line) was calculated from a 30 min EEG recording before the kainic acid treatment. Abbreviations: ISS and CSS: ipsi- and contralateral somatosensory cortex, CFC: contralateral frontal cortex. Scale bars: A = 100 μm, B = 50 μm, C = 300 μV.

In these animals, the 4-AP or pilocarpine injection did not produce *status epilepticus*. Kainic acid was injected on the following day. Despite the possible tolerance effect in these animals that would reduce the numbers of "dark" neurons, the "dark" neurons were consistently found in the PRF. Out of the four rats examined, two received 4-AP a day before. After kainic acid injection, these rats entered *status epilepticus *at 3 h and their PRF contained 15-30 giant "dark" neurons (see Table 1 and Figure 3). Two rats received pilocarpine treatment a day before. After kainic acid injection, one of these rats entered *status epilepticus *at 3 h and had 10-15 "dark" neurons in its PRF. The other rat had multiple seizures without *status epilepticus *and had 4-5 "dark" neurons in its PRF (Table 1).

### EEG analysis

The EEG was examined in eight rats (see ac4_E_, pi4_E_, pi6_E_, ka1-4_E _and ak2_E _in Table 1). Five rats entered *status epilepticus *after their respective treatment. The one-hour power spectral density (PSD) showed a high peak at 1-1.5 Hz in case of focal-cortical crystal treatment (Figure 1G), a prominent peak at 1.5-2.0 Hz and a high increase in power at around 6 Hz in case of pilocarpine treatment (Figure 1H, and see Additional file 2) at 3 hour. From the four kainic acid injected rats as a single treatment two entered *status epilepticus *and they had a prominent peak at 1.5-2.0 Hz in their PSD (see Additional file 2). In case of rat ak2_E_, after its 4-AP i.p. injection, the one-hour PSD showed only a smaller peak at 2 Hz, but after its kainic acid injection there was a prominent, high peak at around 2.0-2.5 Hz (Figure 3D).

## Discussion

In this study, we demonstrated that the pontine reticular formation is affected in *status epilepticus*. It is not completely unknown that the PRF may participate in the generalization and maintenance of epileptic state. Glutamate microinjections into the pons induces electrographic seizures and clonic convulsions [13]. Infusion of NMDA antagonists into the PRF inhibits the generation of tonic and clonic seizures induced by electroshock or pentylenetetrazol [12,14]. PRF neurons are implicated in seizure propagation in several forms of generalized clonic seizures, including audiogenic seizures [15]. Furthermore, human fMRI and SPECT studies have also shown the involvement of the pons in epileptic seizures [21-23].

The injured giant "dark" neurons of the PRF in this study were found by the Gallyas silver staining method. This sensitive method highlights the injured cells in a Golgi-like manner, but does not provide any information about the fate of the highlighted "dark" neurons. On the other hand, it has long been known that the epilepsy-induced "dark" neurons are able to recover [24]. Previous histopathological studies have demonstrated that the compacted "dark" neurons have a high potential for recovery, but this capacity is influenced by the local environment with its (patho-)metabolic processes [25,26]. The "dark" neurons at issue are in an otherwise undamaged environment, so we regard the observed "dark" neurons in the PRF as injured cells with the capacity to recover. Nevertheless, the gigantocellular network of the PRF was affected in our study during *status epilepticus*, independently of the way the *status epilepticus *was induced.

Regarding the functional consequence of injury to the gigantocellular network of PRF, it is well known from the classic discovery of Moruzzi and Magoun, that high-frequency stimulation of the reticular formation produces an arousal response in the cortex. The stimulation is the most effective in bringing about arousal when it is applied to the PRF [27]. Lesions of the reticular formation produce a state of deep sleep or coma, block the arousal, and lead to low frequency/high amplitude activity in the EEG [28,29]. This resembles the slow oscillation developed in our experiments in connection with *status epilepticus*, so we could regard the observed slow oscillation as the electrophysiological consequence of the formation of "dark", compacted, dysfunctional neurons in the PRF.

The observed slow oscillation is not completely unknown in epilepsy research. In kindled animals, delayed slow rhythmic outlasting activities were reported at ~1.5 Hz, which gradually developed following successive acute seizures [30]. The occurrence of interictal slow delta activities were also reported in patients with epilepsy, and the presence of these electric activities was associated with more severe forms of epilepsy [31-33]. Rhythmic diffuse delta frequency activity was also reported in non-convulsive *status epilepticus *[34,35].

Treiman et al. described the distinct EEG patterns that occur during prolonged episodes of *status epilepticus*. They found that these identifiable EEG patterns occur in a predictable order during *status epilepticus*, and the progression of EEG changes during *status epilepticus *follows a common electrical sequence regardless of the initiating factors [2]. The last phase of these patterns was called periodic epileptiform discharges, and it has a low frequency. Later this periodic epileptiform discharges (PEDs) phase was subdivided into early and late PEDs [36]. Early PEDs were characterized by monomorphic periodic discharges with a relatively short flat background at around ~1.5 Hz. Late PEDs showed complex polymorphic periodic discharges with a longer flat background at less than ~1.0 Hz [37]. The slow oscillations in our study would represent the early PEDs, and it is worth noting that significantly elevated glucose utilization rates were observed in the PRF during this phase of *status epilepticus *[36].

Our hypothesis about the generalization of the slow oscillation is the following. The giant neurons of the PRF are receiving intense activation during the repeated epileptic seizures, and their intense activation causes the ascending reticular activating system (ARAS) to promote the generalization of the seizures. The giant neurons of the PRF are glutamatergic, and they innervate each other, sending their long ascending projections primarily to the intralaminar nuclei of the thalamus, which in turn project to widespread cortical regions [38]. The seizure-related hyper-excitability of these glutamatergic neurons is reflected in their elevated glucose utilization. The sustained depolarization, on the other hand, results in elevated intracellular Ca^++ ^levels in the PRF and ultimately an injured, compacted state: the formation of "dark" neurons. We think that the giant "dark" neurons are suspending their activity or not responding properly to synaptic (sensory) activation. This will result in a depression of the functioning of the PRF network and because of the depression of the ARAS, generalized, synchronized slow oscillation in the cortical EEG develops [28]. It is notable that the observed slow oscillation, and especially the late PEDs phases of the *status epilepticus *are resemble to the 'slow' neocortical oscillation described by Steriade [39,40].

Generalized slow neocortical activity is also associated with EEG changes during the state of coma [29,41]. This could be important, as experimental animals in prolonged *status epilepticus *could also enter post-ictal coma before full recovery [42,43]. Lesions in the PRF are associated with brainstem coma [29], but lesions in the reticular formation have been found in brains of people who have post-polio fatigue syndrome [44,45]. Similarly post-ictal fatigue is one of the symptoms that can help distinguish patients with epileptic seizures from those with non-epileptic seizures [46], and this could also suggest the involvement of the PRF in epileptic seizures.

## Conclusion

We argue that the PRF is affected during *status epilepticus*, and its involvement could be important in the progression of the observed slow oscillation corresponding to the periodic epileptiform discharges phase of *status epilepticus*.

## Methods

### Animals

Twenty-six adult male Sprague-Dawley rats (250-300 g; Charles RiverLaboratories, Hungary) were kept in standard conditions under a 12 h light-dark cycle and food and water were supplied *ad libitum*. The experiments were carried out on the basis of local ethical rules in accordance with the Hungarian Act of Animal Care and Experimentation (1998, XXVIII, section 243/1998), which conforms to the regulation of animal experiments in the European Community. All efforts were made to minimize pain and suffering and to reduce the number of animals used.

### Focal-cortical 4-AP application

Rats (n = 5) were anesthetized with a 1-1.5% halothane (Narcotan, Leciva, Praha, Czech Republic)-air mixture and secured in a stereotaxic frame (David Kopf, USA). For the focal 4-AP application, a hole (1.5 mm in diameter) was drilled into the skull above the right parieto-occipital cortex (A: 6.2 mm, L: 2.5 mm) [47]. The dura mater was carefully removed, and a piece of 4-AP crystal (0.5 mg/kg, Sigma-Aldrich, Hungary) was locally placed onto the cortex. The hole was covered with a piece of artificial fibrin sponge (Spongostan, Hungaropharma, Budapest, Hungary). Forty minutes thereafter, the hole was washed out with physiological saline, covered with bone wax (Medicommers Kft., Budapest, Hungary), and the halothane anesthesia was discontinued. Four rats were examined histopathologically, out of the five receiving this treatment. One rat died at 2-h, and it was not examined.

### Systemic injections of 4-AP, pilocarpine and kainic acid

Seizures were induced by systemic injections of 4-AP (4-amionpyridine; Sigma-Aldrich, Hungary, 4.5-5 mg/kg; n = 7, i.p.), pilocarpine (pilocarpine hydrochloride; Sigma-Aldrich, Hungary, 340-370 mg/kg; n = 6, i.p.) or kainic acid (kainic acid, Sigma-Aldrich, Hungary, 10 mg/kg; n = 4, s.c.). Approximately 10 min after 4-AP, pilocarpine or kainic acid injections the animals had seizures and in about 1.5 hours most of the animals entered *status epilepticus*. During the 4-AP i.p. experiments, three rats died soon after receiving the treatment, and they were not examined histopathologically.

### Systemic injections of kainic acid as a second treatment after 4-AP or pilocarpine

As a cumulative approach to bring about *status epilepticus *when the others failed, kainic acid was also injected as a second treatment. The kainic acid treatment was performed on 4 animals that received 4-AP (n = 2) or pilocarpine (n = 2) injections a day before, but did not produce *status epilepticus*. Kainic acid (kainic acid, Sigma-Aldrich, Hungary, 10 mg/kg; n = 4) was injected subcutaneously on the following day, and in about 1.5 hours most of the animals entered *status epilepticus*.

### Behavioral scoring

To score the behavior of the rats after the induction of epileptic seizures, the Racine Scale was used [48] with modifications [49,50]. Specifically, Stage-0: no response; Stage-1: behavioral arrest (motionless), hair raising and rapid breathing salivation or hyperactivity, restlessness and vibrissae twitching (movement of the lips, tongue and vibrissae); Stage-2: head nodding, head and eye clonus (myoclonic jerks); Stage-3: forelimb clonus (unilateral or bilateral limb clonus) and "wet dog shakes"; Stage-4: clonic seizures (forelimb clonic seizures) and clonic rearing; Stage-5: generalized clonic seizures with falling, uncontrollable jumping, and in the later phase, atonia.

### EEG recordings

In eight rats (4-AP crystal, pilocarpine and kainic acid), EEG recordings were also made before the histology. In these animals, one week prior to the induction of the epileptic seizures, under halothane anesthesia, six holes were drilled into the superficial layer of the skull above the frontal, the somatosensory and the parieto-occipital cortices bilaterally; each was filled with conductive paste, through which a stainless-steel electrode was inserted. The electrodes and the connector were embedded in dental acrylic. This way the electrodes were firmly fixed to the skull but the dura were not pierced. In five animals, 2-3 hours prior to the induction of the epileptic seizures, under halothane anesthesia, six EEG electrodes were firmly clipped to the bare skin above the skull in similar positions as above. These electrodes and the connector were also embedded in dental acrylic. The EEG activity was recorded by a Grass EEG 8B model (Grass Instruments, Quincy, MA, USA), filtered at 0.3 Hz to 70 Hz and amplified (20 k). Data was recorded with a CED 1401 system using SPIKE2 v2.1 software (Cambridge Electronic Design Limited, Cambridge, UK). The sampling rate was 3000 Hz. From the 60-minute records the power spectral density (PSD) was calculated (NeuroExplorer v.3.2, Nex Technologies, MA, USA). The specific parameters used were: Max. Freq. (Hz) = 10 Hz; Number of fr. values = 512; Interval filter = None; Smooth = None.

The five identifiable EEG phases, which occur during the course of the GCSE were: (1) discrete seizures; (2) merging seizures with waxing and waning amplitude and frequency of EEG rhythms; (3) continuous ictal activity; (4) continuous ictal activity punctuated by low voltage 'flat periods'; and (5) periodic epileptiform discharges on a 'flat' background [2].

### Perfusion and tissue sectioning

Rats were deeply anesthetized with an overdose of urethane (2 g/kg i.p., Sigma-Aldrich, Hungary), and perfused through the aorta with physiological saline followed by 4% buffered paraformaldehyde [11]. Brains were removed from the skull 1 day later, then immersed in the same fixative for 1-3 days and frozen-sectioned at 60 μm.

### Silver staining of "dark" neurons

Sections were incubated for 16 h at 56°C (esterification) in 1-propanol (Reanal, Budapest, Hungary) containing 1.2% sulfuric acid (Carlo Erba Reagents, Italy). Following a 5-minute treatment in 1% acetic acid (Reanal, Budapest, Hungary), they were immersed in a silicotungstate physical developer until the background turned yellowish-brown [11]. Development was terminated by washing in 1% acetic acid for 30 min. Sections were dehydrated, mounted, embedded in DePex (Fluka, Hungary) and coverslipped.

## List of abbreviations

PRF: pontine reticular formation; GCSE: generalized convulsive *status epilepticus*; 4-AP: 4-aminopyridine; SE: *status epilepticus*; PnO: oral and pontine reticular field; PnC: caudal pontine reticular field; Gi: medullary reticular field; NMDA: N-methyl-D-aspartic acid; PSD: power spectral density; PEDs: periodic epileptiform discharges; ARAS: ascending reticular activating system.

## Authors' contributions

BP conceived of the study and carried out the experiments. VK participated in the experiments. KAK participated in the design of the study and coordination. GJ participated in the design of the study, coordination and helped to draft the manuscript. AC conceived of the study, participated in its design and drafted the manuscript. All authors read and approved the final manuscript.

## Supplementary Material

Additional file 1 Negativ control (A) and giant „dark” neurons (B) in the oral part of the pontine reticular formation, 3 hours after systemic injection of pilocarpine. Scale bars: 200 μm. Click here for file

Additional file 2 Representative EEG periods demonstrate the generalized convulsive *status epilepticus *after pilocarpine (A) and kainic acid (C and E) injections. One-hour PSD graphs show high peaks at the slow frequencies (1.5-2 Hz) after pilocarpine (B) and kainic acid injection (D and F). Scale bars: A, C and E = 200 μV. Click here for file

## References

[B1] Drislane FW Who's afraid of status epilepticus? Epilepsia 2006 47 1 7 9 10.1111/j.1528-1167.2006.00363.x 16417525

[B2] Treiman DM Walton NY Kendrick C A progressive sequence of electroencephalographic changes during generalized convulsive status epilepticus Epilepsy Res 1990 5 1 49 60 10.1016/0920-1211(90)90065-4 2303022

[B3] Treiman DM Electroclinical features of status epilepticus J Clin Neurophysiol 1995 12 4 343 62 7560022

[B4] Mello LE Covolan L Spontaneous seizures preferentially injure interneurons in the pilocarpine model of chronic spontaneous seizures Epilepsy Res 1996 26 1 123 9 10.1016/S0920-1211(96)00048-4 8985694

[B5] Poirier JL Capek R De Koninck Y Differential progression of Dark Neuron and Fluoro-Jade labelling in the rat hippocampus following pilocarpine-induced status epilepticus Neuroscience 2000 97 1 59 68 10.1016/S0306-4522(00)00026-9 10771339

[B6] Covolan L Mello LE Temporal profile of neuronal injury following pilocarpine or kainic acid-induced status epilepticus Epilepsy Res 2000 39 2 133 52 10.1016/S0920-1211(99)00119-9 10759302

[B7] Covolan L Mello LE Assessment of the progressive nature of cell damage in the pilocarpine model of epilepsy Braz J Med Biol Res 2006 39 7 915 24 10.1590/S0100-879X2006000700010 16862283

[B8] Baracskay P Szepesi Z Orban G Juhasz G Czurko A Generalization of seizures parallels the formation of "dark" neurons in the hippocampus and pontine reticular formation after focal-cortical application of 4-aminopyridine (4-AP) in the rat Brain Res 2008 1228 217 28 10.1016/j.brainres.2008.06.044 18602900

[B9] Soderfeldt B Kalimo H Olsson Y Siesjo BK Bicuculline-induced epileptic brain injury. Transient and persistent cell changes in rat cerebral cortex in the early recovery period Acta Neuropathol 1983 62 1-2 87 95 10.1007/BF00684924 6659880

[B10] Gallyas F Guldner FH Zoltay G Wolff JR Golgi-like demonstration of "dark" neurons with an argyrophil III method for experimental neuropathology Acta Neuropathol (Berl) 1990 79 6 620 8 10.1007/BF00294239 1694382

[B11] Gallyas F Hsu M Buzsaki G Four modified silver methods for thick sections of formaldehyde-fixed mammalian central nervous tissue: 'dark' neurons, perikarya of all neurons, microglial cells and capillaries J Neurosci Methods 1993 50 2 159 64 10.1016/0165-0270(93)90004-B 8107497

[B12] Peterson SL Infusion of NMDA antagonists into the nucleus reticularis pontis oralis inhibits the maximal electroshock seizure response Brain Res 1995 702 1-2 101 9 10.1016/0006-8993(95)01026-2 8846064

[B13] Elazar Z Berchanski A Excitatory amino acids modulate epileptogenesis in the brain stem Neuroreport 2000 11 8 1777 80 10.1097/00001756-200006050-00036 10852243

[B14] Manjarrez J Alvarado R Camacho-Arroyo I Differential effects of NMDA antagonists microinjections into the nucleus reticularis pontis caudalis on seizures induced by pentylenetetrazol in the rat Epilepsy Res 2001 46 1 39 44 10.1016/S0920-1211(01)00256-X 11395287

[B15] Raisinghani M Faingold CL Pontine reticular formation neurons are implicated in the neuronal network for generalized clonic seizures which is intensified by audiogenic kindling Brain Res 2005 1064 1-2 90 7 10.1016/j.brainres.2005.09.047 16336948

[B16] Blondeau N Widmann C Lazdunski M Heurteaux C Activation of the nuclear factor-kappaB is a key event in brain tolerance J Neurosci 2001 21 13 4668 77 11425894 10.1523/JNEUROSCI.21-13-04668.2001 PMC6762345

[B17] Boeck CR Ganzella M Lottermann A Vendite D NMDA preconditioning protects against seizures and hippocampal neurotoxicity induced by quinolinic acid in mice Epilepsia 2004 45 7 745 50 10.1111/j.0013-9580.2004.65203.x 15230696

[B18] Marini AM Jiang X Wu X Pan H Guo Z Mattson MP Blondeau N Novelli A Lipsky RH Preconditioning and neurotrophins: a model for brain adaptation to seizures, ischemia and other stressful stimuli Amino Acids 2007 32 3 299 304 10.1007/s00726-006-0414-y 16998712

[B19] Hatazaki S Bellver-Estelles C Jimenez-Mateos EM Meller R Bonner C Murphy N Matsushima S Taki W Prehn JH Simon RP Henshall DC Microarray profile of seizure damage-refractory hippocampal CA3 in a mouse model of epileptic preconditioning Neuroscience 2007 150 2 467 77 17935890 10.1016/j.neuroscience.2007.09.020 PMC2268097

[B20] Ogita K Okuda H Watanabe M Nagashima R Sugiyama C Yoneda Y In vivo treatment with the K+ channel blocker 4-aminopyridine protects against kainate-induced neuronal cell death through activation of NMDA receptors in murine hippocampus Neuropharmacology 2005 48 6 810 21 10.1016/j.neuropharm.2004.12.018 15829253

[B21] Federico P Archer JS Abbott DF Jackson GD Cortical/subcortical BOLD changes associated with epileptic discharges: an EEG-fMRI study at 3 T Neurology 2005 64 7 1125 30 15824333 10.1212/01.WNL.0000156358.72670.AD

[B22] Tae WS Joo EY Han SJ Lee KH Hong SB CBF changes in drug naive juvenile myoclonic epilepsy patients J Neurol 2007 254 8 1073 80 10.1007/s00415-006-0491-6 17351720

[B23] Joo EY Tae WS Hong SB Regional effects of lamotrigine on cerebral glucose metabolism in idiopathic generalized epilepsy Arch Neurol 2006 63 9 1282 6 10.1001/archneur.63.9.1282 16966506

[B24] Atillo A Soderfeldt B Kalimo H Olsson Y Siesjo BK Pathogenesis of brain lesions caused by experimental epilepsy. Light- and electron-microscopic changes in the rat hippocampus following bicuculline-induced status epilepticus Acta Neuropathol 1983 59 1 11 24 10.1007/BF00690312 6301201

[B25] Csordas A Mazlo M Gallyas F Recovery versus death of "dark" (compacted) neurons in non-impaired parenchymal environment: light and electron microscopic observations Acta Neuropathol (Berl) 2003 106 1 37 49 10.1007/s00401-003-0694-1 12665989

[B26] Gallyas F Gasz B Szigeti A Mazlo M Pathological circumstances impair the ability of "dark" neurons to undergo spontaneous recovery Brain Res 2006 1110 1 211 20 10.1016/j.brainres.2006.06.078 16872583

[B27] Moruzzi G Magoun HW Brain stem reticular formation and activation of the EEG Electroencephalogr Clin Neurophysiol 1949 1 4 455 73 18421835

[B28] Camacho Evangelista A Reinoso Suarez F Activating and Synchronizing Centers in Cat Brain: Electroencephalograms after Lesions Science 1964 146 268 70 10.1126/science.146.3641.268 14185325

[B29] Parvizi J Damasio AR Neuroanatomical correlates of brainstem coma Brain 2003 126 Pt 7 1524 36 10.1093/brain/awg166 12805123

[B30] Nita DA Cisse Y Timofeev I State-dependent slow outlasting activities following neocortical kindling in cats Exp Neurol 2008 211 2 456 68 10.1016/j.expneurol.2008.02.010 18420200

[B31] Koutroumanidis M Martin-Miguel C Hennessy MJ Akanuma N Valentin A Alarcon G Jarosz JM Polkey CE Interictal temporal delta activity in temporal lobe epilepsy: correlations with pathology and outcome Epilepsia 2004 45 11 1351 67 10.1111/j.0013-9580.2004.61203.x 15509236

[B32] Hughes JR Fino JJ EEG in seizure prognosis: association of slow wave activity and other factors in patients with apparent misleading epileptiform findings Clin EEG Neurosci 2004 35 4 181 4 15493532 10.1177/155005940403500407

[B33] Hughes JR Fino JJ Kaydanova Y The EEG profile of patients with uncontrolled vs. controlled seizures Clin EEG Neurosci 2004 35 2 69 77 15164813 10.1177/155005940403500204

[B34] Vinton A Kornberg AJ Cowley M Matkovic Z Kilpatrick C O'Brien TJ Tiagabine-induced generalised non convulsive status epilepticus in patients with lesional focal epilepsy J Clin Neurosci 2005 12 2 128 33 10.1016/j.jocn.2004.03.027 15749411

[B35] Uthman B Bearden S Rhythmic diffuse delta frequency activity presenting as an unusual EEG correlate of nonconvulsive status epilepticus: three case studies Epilepsy Behav 2008 12 1 191 9 10.1016/j.yebeh.2007.08.019 17950037

[B36] Handforth A Treiman DM Functional mapping of the late stages of status epilepticus in the lithium-pilocarpine model in rat: a 14C-2-deoxyglucose study Neuroscience 1995 64 4 1075 89 10.1016/0306-4522(94)00377-H 7753376

[B37] Jung KY Kim JM Kim DW Nonlinear dynamic characteristics of electroencephalography in a high-dose pilocarpine-induced status epilepticus model Epilepsy Res 2003 54 2-3 179 88 10.1016/S0920-1211(03)00079-2 12837569

[B38] Jones BE Paxinos GT Reticular Formation: Cytoarchitecture, Transmitters, and Projection The Rat Nervous System 1994 1 San Diego, Calif: Academic Press 155 171

[B39] Steriade M McCormick DA Sejnowski TJ Thalamocortical oscillations in the sleeping and aroused brain Science 1993 262 5134 679 85 10.1126/science.8235588 8235588

[B40] Steriade M Nunez A Amzica F A novel slow (< 1 Hz) oscillation of neocortical neurons in vivo: depolarizing and hyperpolarizing components J Neurosci 1993 13 8 3252 65 8340806 10.1523/JNEUROSCI.13-08-03252.1993 PMC6576541

[B41] Loeb C Electroencephalographic changes during the state of coma Electroencephalogr Clin Neurophysiol 1958 10 4 589 606 10.1016/0013-4694(58)90061-0 13597808

[B42] Turski L Ikonomidou C Turski WA Bortolotto ZA Cavalheiro EA Review: cholinergic mechanisms and epileptogenesis. The seizures induced by pilocarpine: a novel experimental model of intractable epilepsy Synapse 1989 3 2 154 71 10.1002/syn.890030207 2648633

[B43] Curia G Longo D Biagini G Jones RS Avoli M The pilocarpine model of temporal lobe epilepsy J Neurosci Methods 2008 172 2 143 57 18550176 10.1016/j.jneumeth.2008.04.019 PMC2518220

[B44] Bruno RL Cohen JM Galski T Frick NM The neuroanatomy of post-polio fatigue Arch Phys Med Rehabil 1994 75 5 498 504 8185440

[B45] Bruno RL Sapolsky R Zimmerman JR Frick NM Pathophysiology of a central cause of post-polio fatigue Ann N Y Acad Sci 1995 753 257 75 10.1111/j.1749-6632.1995.tb27552.x 7611635

[B46] Ettinger AB Weisbrot DM Nolan E Devinsky O Postictal symptoms help distinguish patients with epileptic seizures from those with non-epileptic seizures Seizure 1999 8 3 149 51 10.1053/seiz.1999.0270 10356371

[B47] Paxinos G Watson C The rat brain in stereotaxic coordinates 1982 Sydney, Academic Press 10.1016/0165-0270(80)90021-7 6110810

[B48] Racine RJ Modification of seizure activity by electrical stimulation. II. Motor seizure Electroencephalogr Clin Neurophysiol 1972 32 3 281 94 10.1016/0013-4694(72)90177-0 4110397

[B49] Malhotra J Gupta YK Effect of adenosine receptor modulation on pentylenetetrazole-induced seizures in rats Br J Pharmacol 1997 120 2 282 8 9117121 10.1038/sj.bjp.0700869 PMC1564359

[B50] Medina-Ceja L Cordero-Romero A Morales-Villagran A Antiepileptic effect of carbenoxolone on seizures induced by 4-aminopyridine: a study in the rat hippocampus and entorhinal cortex Brain Res 2008 1187 74 81 10.1016/j.brainres.2007.10.040 18031716

