# Supplementary figures and images for: Status epilepticus affects the gigantocellular network of the pontine reticular formation
Source: BMC Neurosci. 2009 Nov 13;10:133. doi: 10.1186/1471-2202-10-133 (PMC2781816; doi:10.1186/1471-2202-10-133)

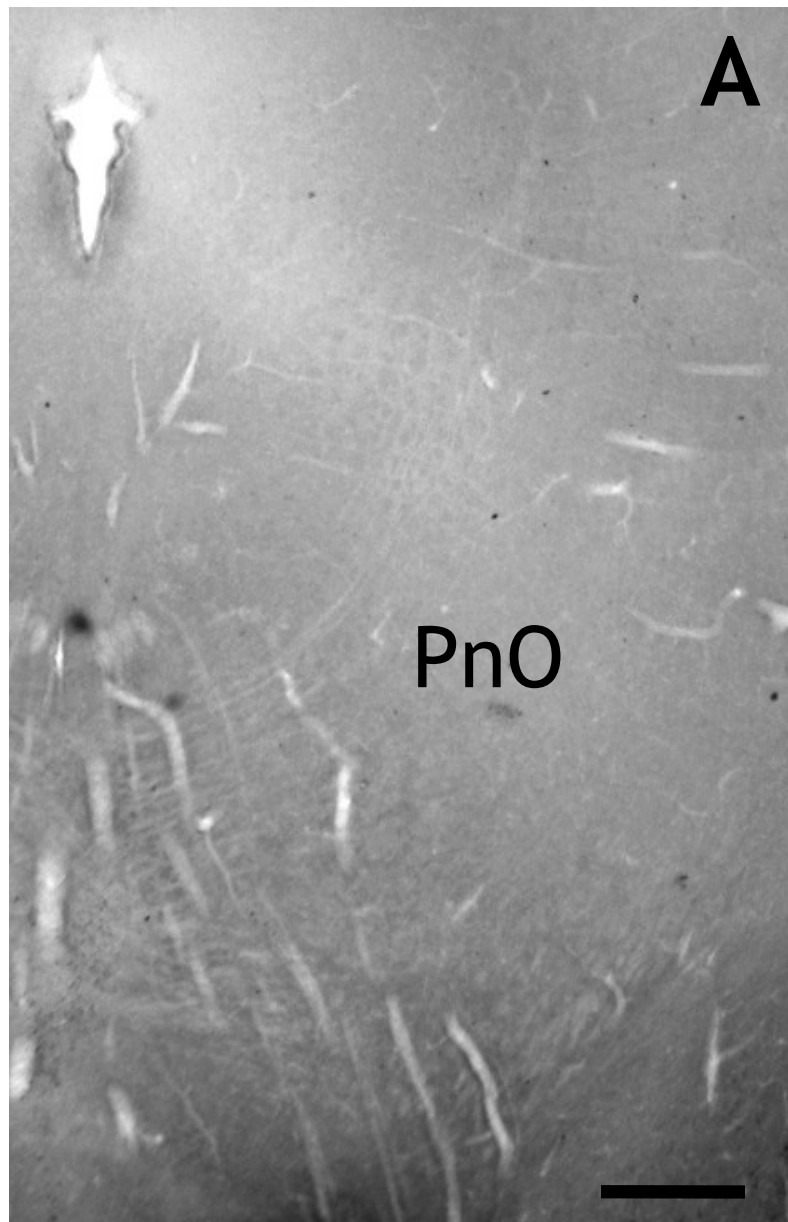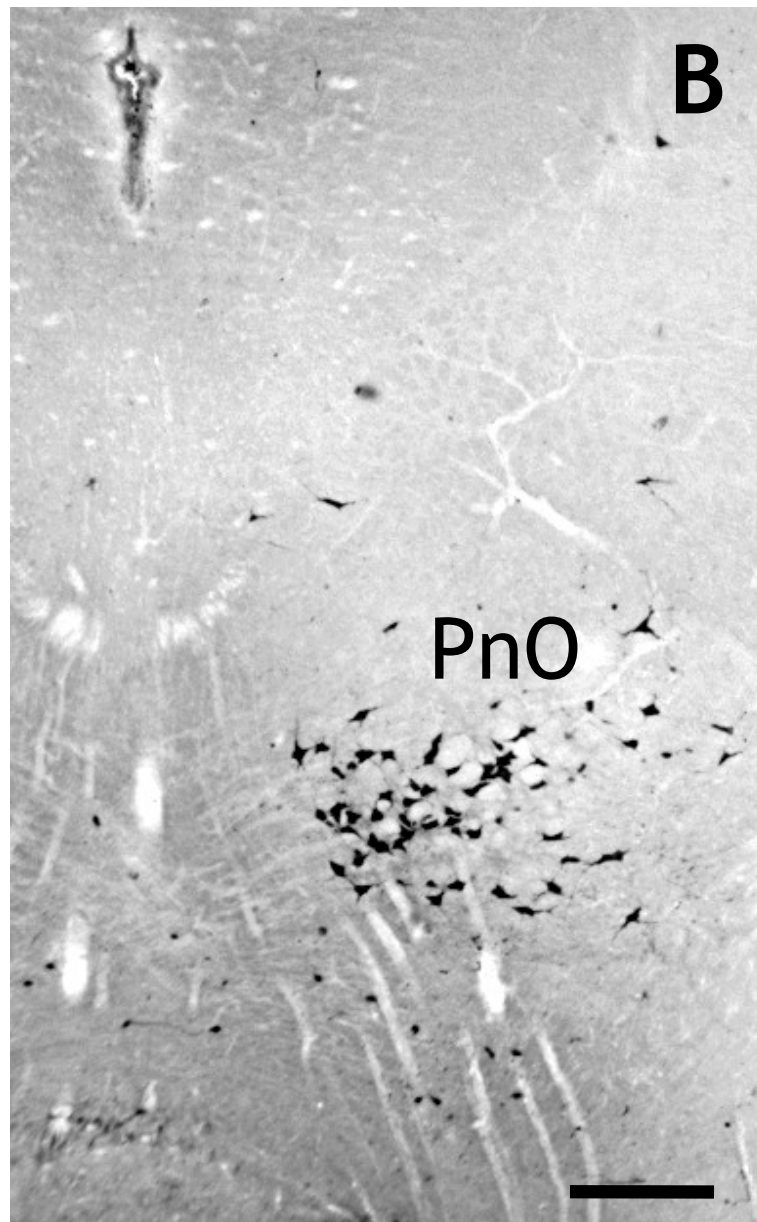

Supplement: Additional file 1 — Negativ control (A) and giant „dark” neurons (B) in the oral part of the pontine reticular formation, 3 hours after systemic injection of pilocarpine. Scale bars: 200 μm. [file 1471-2202-10-133-S1.pdf]

**A**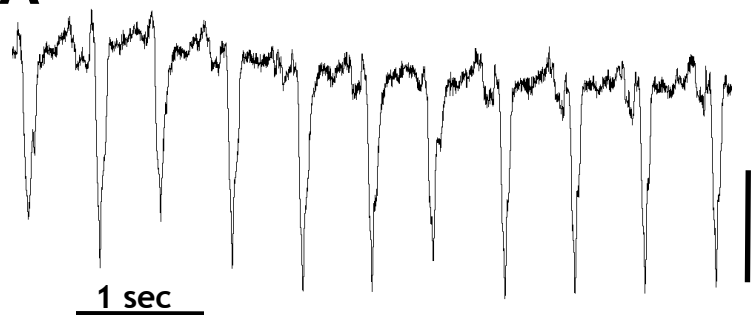**B**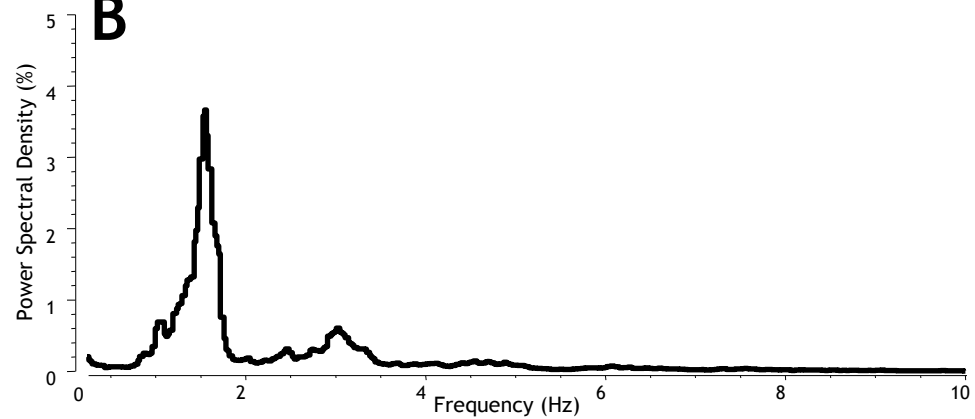**C**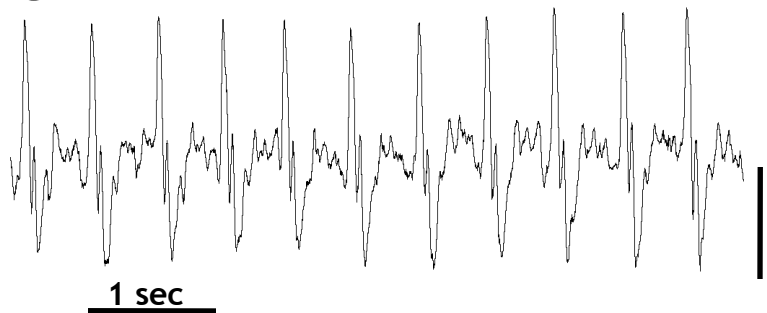**D**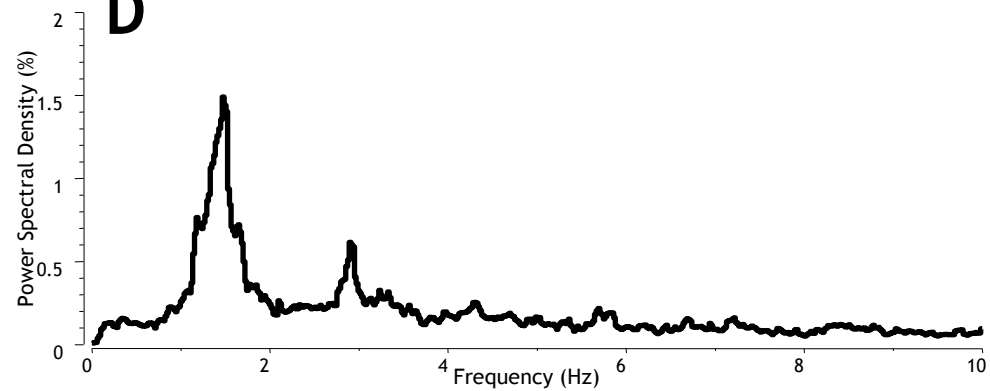**E**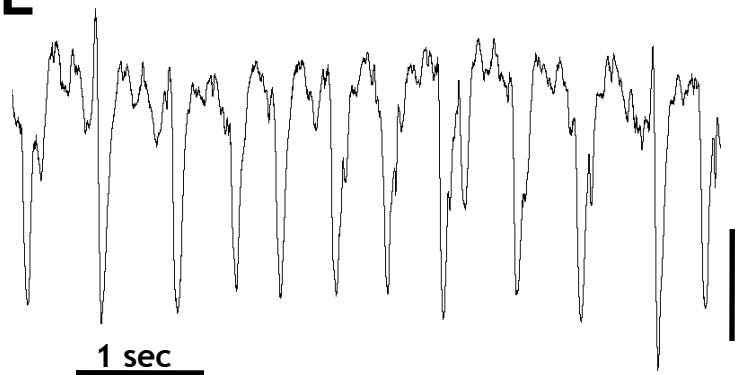**F**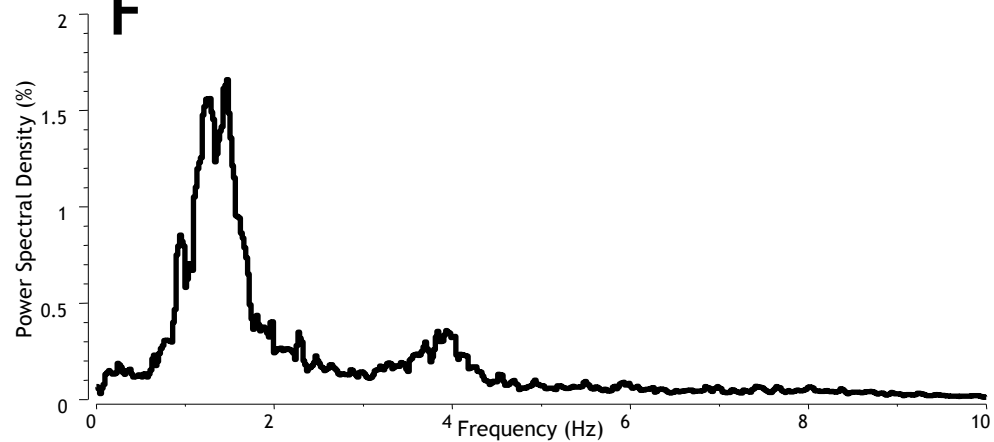

Supplement: Additional file 2 — Representative EEG periods demonstrate the generalized convulsive status epilepticus after pilocarpine (A) and kainic acid (C and E) injections. One-hour PSD graphs show high peaks at the slow frequencies (1.5-2 Hz) after pilocarpine (B) and kainic acid injection (D and F). Scale bars: A, C and E = 200 μV. [file 1471-2202-10-133-S2.pdf]
